# GRWD1 enhances HSV-1 replication by facilitating nuclear egress

**DOI:** 10.1128/spectrum.01608-25

**Published:** 2026-04-17

**Authors:** Zimeng Kong, Chao Gao, Xingyun Liu, Teng Xue, Xinxin Liang, Peijie Yan, Rui Zhang, Jun Tang

**Affiliations:** 1 National Key Laboratory of Veterinary Public Health Security, Key Laboratory of Animal Epidemiology of the Ministry of Agriculture and Rural Affairs, College of Veterinary Medicine, China Agricultural University 630101 , Beijing, China; Oklahoma State University College of Veterinary Medicine, Stillwater, Oklahoma, USA

**Keywords:** GRWD1, HSV-1 replication, nuclear egress, US3, Lamin A/C

## Abstract

Herpes simplex virus 1 (HSV-1) depends on host factors for its replication. Here, we identify glutamate-rich WD40 repeat-containing 1 (GRWD1) as a novel pro-viral host factor that facilitates HSV-1 nuclear egress. GRWD1 knockdown trapped nucleocapsids in the nucleus and suppressed viral replication. This function appears to require the viral kinase US3, as GRWD1’s effect was abolished in US3-deficient infections. We further show that GRWD1 interacts with US3 and partially colocalizes with the viral nuclear egress complex protein UL34 at the nuclear membrane in both HSV-1-infected and HSV-1ΔUS3-infected cells. Mechanistically, GRWD1 promotes the proteasomal degradation of Lamin A/C and directly binds Lamin A, suggesting a model where it disrupts the nuclear lamina to facilitate capsid exit. Our findings reveal a novel virus-host interaction that promotes HSV-1 nuclear egress.

**IMPORTANCE:**

Alphaherpesviruses, including herpes simplex virus 1 (HSV-1), cause lifelong infections and severe diseases globally by subverting host machinery for replication. Using HSV-1 as a model, we identified glutamate-rich WD40 repeat-containing 1 (GRWD1) as a critical host factor enabling viral nuclear egress, a key step in viral propagation. GRWD1 facilitates nuclear escape of capsids via degradation of Lamin A/C and directly interacts with Lamin A. These findings reveal a novel mechanism by which HSV-1 exploits GRWD1 to breach nuclear barriers, highlighting GRWD1 as a potential target for disrupting herpesvirus replication and developing broad-spectrum antivirals.

## INTRODUCTION

Herpes simplex virus 1 (HSV-1), an alphaherpesvirus, significantly impacts human health globally (1, 2). Its life cycle encompasses adsorption and entry via virion envelope protein and cell surface receptor interactions, followed by membrane fusion or endocytosis (3, 4). The capsid is then transported to the nuclear pore via the cytoskeleton (5). Inside the nucleus, the progeny genome replicates and assembles into nucleocapsids (6–8). These nucleocapsids bud through the inner nuclear membrane (INM) for primary envelopment, then fuse with the outer nuclear membrane for de-envelopment, releasing them into the cytoplasm (9, 10). Tegumentation and secondary envelopment occur in the trans-Golgi network or endosome, generating mature virions (11), which exit via exocytosis or cell-to-cell spread (12).

A critical step in the HSV-1 life cycle is nuclear egress, during which nucleocapsids must traverse the nuclear lamina to reach the INM. The nuclear lamina, composed of Lamin A/C (derived from RNA splicing variants of the LMNA transcript [13]), Lamin B1, Lamin B2, emerin, and associated proteins, forms a fibrous network lining the nucleoplasmic surface of the INM. This structure provides structural support to the nucleus and regulates transcription and DNA replication (14). It also functions as a barrier to efficient egress, the breaching of which involves remodeling of the lamina, a process driven by the interplay between viral components and host nuclear architecture that is not fully understood.

The HSV-1 kinase US3 plays a pivotal role in regulating nuclear egress (15). In the absence of US3, nucleocapsids accumulate in the perinuclear space (16, 17), disrupting egress. However, US3 is not essential for alphaherpesvirus replication, as nuclear egress can still occur without it, albeit at a slower rate (18). US3 facilitates this process by phosphorylating Lamin A/C (19) and the core nuclear egress complex (NEC) components UL31 and UL34, and it likely targets additional host proteins to further optimize nuclear egress (20). These phosphorylation events are thought to destabilize the lamina and enhance NEC function, ensuring smooth nucleocapsid exit.

Glutamate-rich WD40 repeat-containing 1 (GRWD1) is a poorly studied host protein. It binds histone to regulate chromatin dynamics and minichromosome maintenance complex loading at replication origins (21) and may play a role in ribosome biogenesis (22, 23). Notably, GRWD1 knockout demonstrates embryonic lethality in zebrafish (24). Additionally, GRWD1 inhibits p53 through the RPL11–MDM2 pathway (25, 26), promoting tumorigenesis, and interacts with DDB1, potentially acting as a substrate receptor for the CUL4-DDB1 ubiquitin ligase complex (27). GRWD1 has also been predicted as a binding partner of Bcl-2-associated transcription factor 1 (Bclaf1) (BioGRID, https://thebiogrid.org/). Our previous discovery showed that HSV-1 hijacks the viral kinase US3 to trigger proteasomal degradation of Bclaf1 (28), while subsequent studies suggest that GRWD1 may act as an E3 ubiquitin ligase component mediating US3-phosphorylation-dependent Bclaf1 degradation. Despite these diverse functions, there is currently no research on GRWD1 in the context of HSV-1 or other viruses.

In this study, we explore the role of GRWD1 in HSV-1 replication, demonstrating that its promotion of viral replication is US3-dependent. GRWD1 knockdown significantly inhibits nuclear egress. Mechanistically, GRWD1 facilitates Lamin A/C degradation through the proteasomal pathway, though whether US3 participates in this process to regulate nuclear egress remains unclear. During HSV-1 infection, GRWD1 relocates from the nucleolus to perinuclear cytoplasmic clusters. These findings establish GRWD1 as a novel host factor in nuclear egress, illuminating a previously unrecognized host-virus interaction.

## RESULTS

### GRWD1 enhances HSV-1 replication

**Fig 1:**
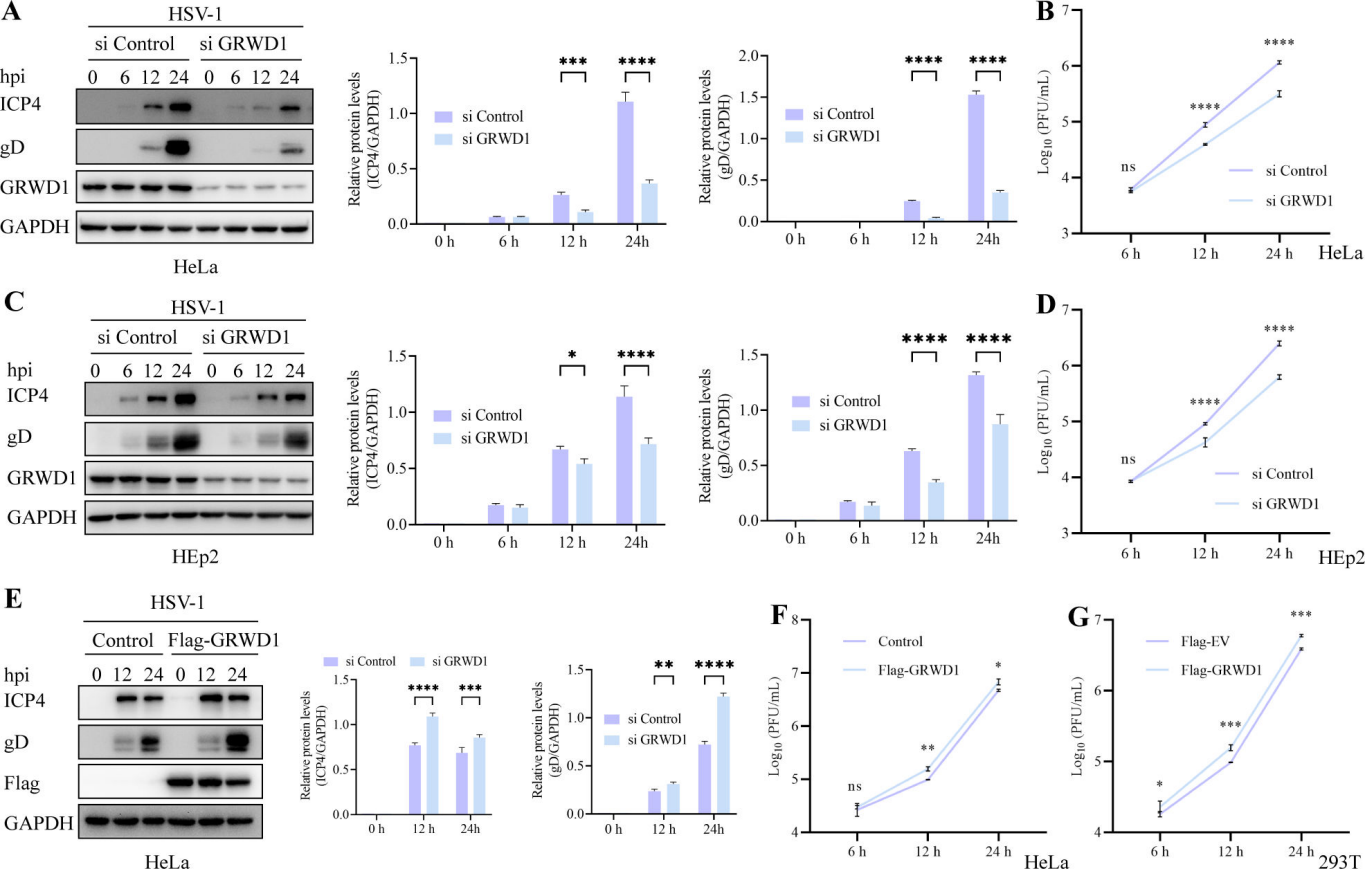
GRWD1 enhances HSV-1 replication. (**A**) WB analysis of viral proteins and GRWD1 in HeLa cells transfected with si Control or si GRWD1 for 48 h, followed by HSV-1 infection (MOI = 1) for the indicated times. GAPDH served as a loading control. Quantification of ICP4 and gD band intensities (densitometric analysis by ImageJ) normalized to GAPDH is shown to the right of the WB. (**B**) Infectious viral titers in the infected cultures of HeLa cells treated as in panel **A**. (**C**) WB analysis of viral proteins and GRWD1 in HEp2 cells under the same conditions as in panel **A**. (**D**) Infectious viral titers in the infected cultures of HEp2 cells treated as in panel **C**. (**E**) WB analysis of viral proteins and GRWD1 in HeLa cells stably expressing Flag-GRWD1 or Flag-empty vector (Control) following HSV-1 infection. (**F**) Infectious viral titers in the infected cultures of Flag-GRWD1 and Control cells treated as in panel **E**. (**G**) Infectious viral titers in the infected cultures of 293T cells transfected with Flag-EV or Flag-GRWD1 for 24 h and then infected with HSV-1 for the indicated times. Statistical analysis: data in panels **A–G** were analyzed by two-way ANOVA followed by Sidak’s multiple comparisons test to evaluate differences between groups at each time point. Asterisks indicate statistical significance between groups at the same time point (*, *P* < 0.05; **, *P* < 0.01; ***, *P* < 0.001; ****, *P* < 0.0001; ns, not significant). Data are presented as mean ± SD from three independent experiments.

To investigate whether GRWD1 plays a role in HSV-1 replication, we transfected HeLa cells with GRWD1 siRNA, which significantly reduced GRWD1 (Fig. 1A and C) without affecting cell viability (data not shown). We then assessed HSV-1 replication in HeLa and HEp2 cells following GRWD1 knockdown. Western blot (WB) analysis showed reduced levels of HSV-1 proteins (ICP4 and gD) after GRWD1 knockdown (Fig. 1A and C). Similarly, viral titer assays (Fig. 1B and D) demonstrated that GRWD1 knockdown significantly reduced HSV-1 replication efficiency, with a 4–5-fold decrease in viral titers compared to controls. To assess the effect of GRWD1 overexpression, we established a HeLa cell line stably overexpressing Flag-GRWD1 (Flag-GRWD1) through lentiviral packaging. Compared with the control cell line (Flag-empty vector [EV] Control HeLa, Control), HSV-1-infected Flag-GRWD1 HeLa cells exhibited moderate increases in viral protein levels (ICP4 and gD) and viral titers (1.5–2-fold increase, Fig. 1E and F). Similarly, transient overexpression of the Flag-GRWD1 plasmid in 293T cells also enhanced HSV-1 titers (Fig. 1G). These results collectively demonstrate that GRWD1 overexpression contributes to HSV-1 replication.

### GRWD1 knockdown impairs HSV-1 nuclear egress

**Fig 2:**
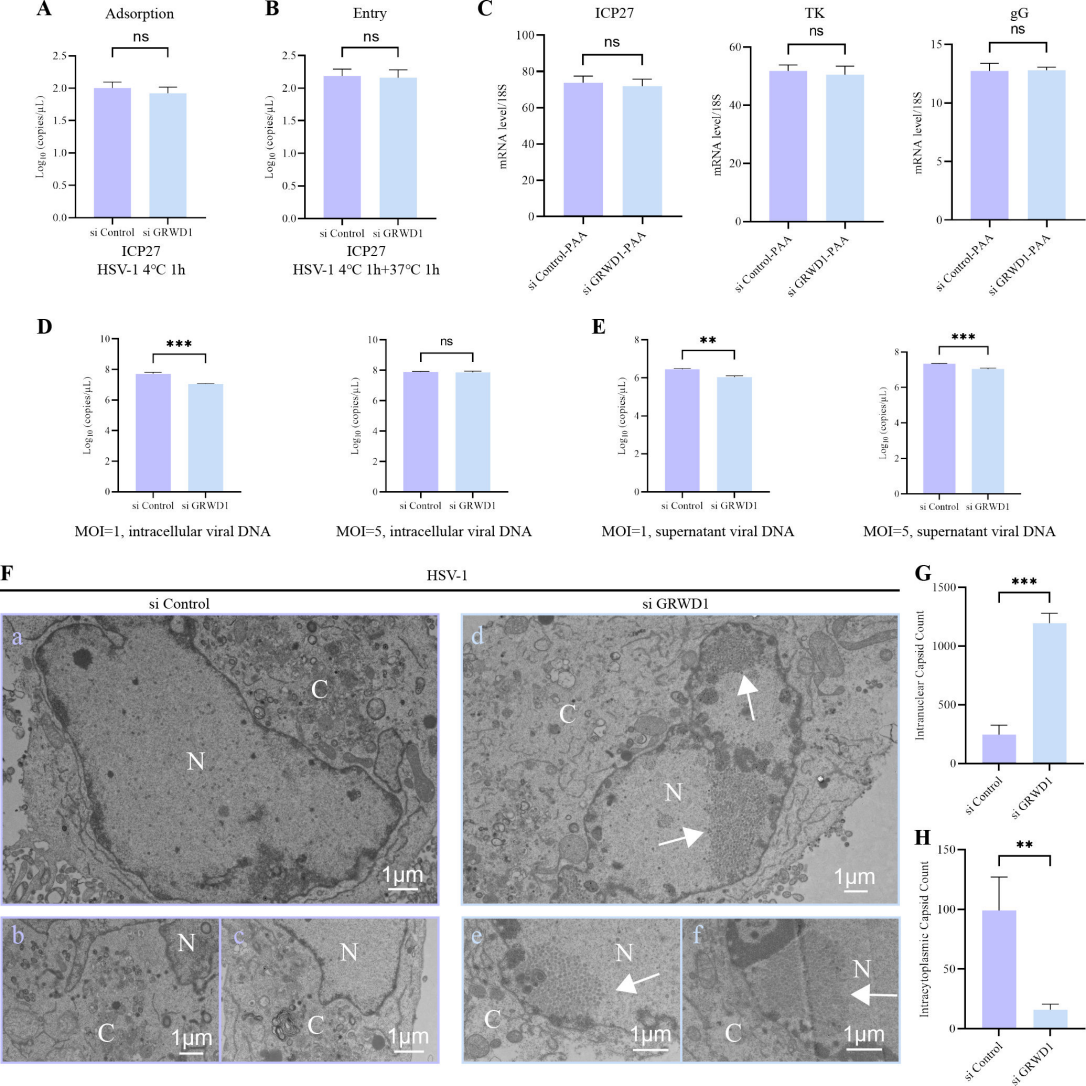
GRWD1 knockdown impairs HSV-1 nuclear egress. (**A**) Viral adsorption assay: HeLa cells pretreated with si Control or si GRWD1 (48 h) were infected with HSV-1 (MOI = 5) and incubated at 4°C for 1 h. (**B**) Viral entry assay: cells pretreated as in panel **A** were adsorbed with HSV-1 (MOI = 5) at 4°C for 1 h and then shifted to 37°C for 1 h. Viral DNA was quantified by qPCR (**A and B**). (**C**) mRNA levels of HSV-1 genes ICP27, TK, and gG in HeLa cells infected with HSV-1 (MOI = 1, 8 hpi) in the presence of PAA (200 ng/mL) added at the onset of infection. Viral genome copy numbers in cell lysates (**D**) or culture supernatants (**E**) from HeLa cells infected with HSV-1 at MOI = 1 or MOI = 5 for 12 h, after si Control or si GRWD1 treatment. Viral DNA was quantified by qPCR. (**F**) Transmission electron microscopy (TEM) of si Control (a–c) or si GRWD1-treated (d–f) HeLa cells infected with HSV-1 (MOI = 1, 24 hpi). N, nucleus; C, cytoplasm. Scale bars: 1 μm. Results from three independent fields. (**G**) Quantification of nuclear viral capsids in GRWD1-knockdown versus control cells. (**H**) Quantification of cytoplasmic viral capsids in GRWD1-knockdown versus control cells. Statistical analysis: panels **A–E**, **G, and H**: unpaired two-tailed Student’s *t*-test. Data are shown as mean ± SD from three independent experiments. Significance levels: **, *P* < 0.01; ***, *P* < 0.001; ns, not significant.

Given the positive regulatory role of GRWD1 in HSV-1 replication, we investigated its impact on distinct stages of the viral lifecycle. First, we examined the effect of GRWD1 knockdown on HSV-1 virion adsorption and entry by performing absolute quantification of viral genome copies through qPCR targeting the ICP27 gene, to evaluate HSV-1 adsorption and entry efficiency under GRWD1-knockdown conditions. No significant differences were observed between GRWD1-knockdown HeLa cells and control cells during early infection stages (Fig. 2A and B). Subsequent analysis of viral gene transcription, including immediate-early gene (ICP27), early gene (TK), and late gene (gG), under phosphonoacetic acid (PAA) treatment showed no significant alterations in GRWD1-depleted cells compared to controls (Fig. 2C). Western blot analysis (Fig. 1) demonstrated that GRWD1’s regulatory effects on viral proteins manifested during mid-to-late infection stages. These findings directed our investigation toward HSV-1 replication/post-replication processes.

To further evaluate the effect of GRWD1 knockdown on HSV-1 genome accumulation, viral DNA copies were quantified in cells and culture supernatants under different MOI conditions. At 12 hpi, GRWD1 depletion led to a significant reduction of intracellular viral genome copies under MOI = 1 infection, with approximately a 5–7-fold decrease compared with the control group (Fig. 2D). Consistently, under MOI = 1 infection, intracellular viral genome copies were comparable at 6 hpi but reduced at 24 hpi after GRWD1 knockdown, as shown in Fig. S1A, supporting the decrease observed at 12 hpi. In contrast, under MOI = 5 infection, intracellular viral genome copies showed no significant difference after GRWD1 knockdown (Fig. 2D), which was consistent with the result at 6 hpi under the same MOI condition (Fig. S1B). For extracellular viral genome copies in culture supernatants, GRWD1 knockdown resulted in a marked decrease at 12 hpi under both MOI = 1 and MOI = 5 infection conditions (Fig. 2E). This decrease was also observed at 24 hpi under MOI = 1 infection (Fig. S1C) and at 6 hpi under MOI = 5 infection (Fig. S1D), in agreement with the reductions detected at 12 hpi. These results indicate that GRWD1 knockdown selectively affects HSV-1 genome accumulation during the mid-to-late phase of infection, suggesting that GRWD1 may be involved in a specific post-replication step of the viral life cycle. To further investigate the underlying functional process, we next examined the ultrastructural features of infected cells by transmission electron microscopy.

Ultrastructural analysis based on three representative cellular fields (Fig. 2F) revealed a clear nuclear retention of viral capsids following GRWD1 knockdown. Quantification from nine randomly selected nuclear fields, irrespective of capsid type, confirmed that GRWD1 depletion significantly increased nuclear capsid numbers (Fig. 2G), whereas cytoplasmic nucleocapsids were significantly more abundant in control cells (Fig. 2H). Collectively, these findings demonstrate that GRWD1 specifically modulates HSV-1 nuclear egress.

### GRWD1’s role in HSV-1 nuclear egress is dependent on US3

**Fig 3:**
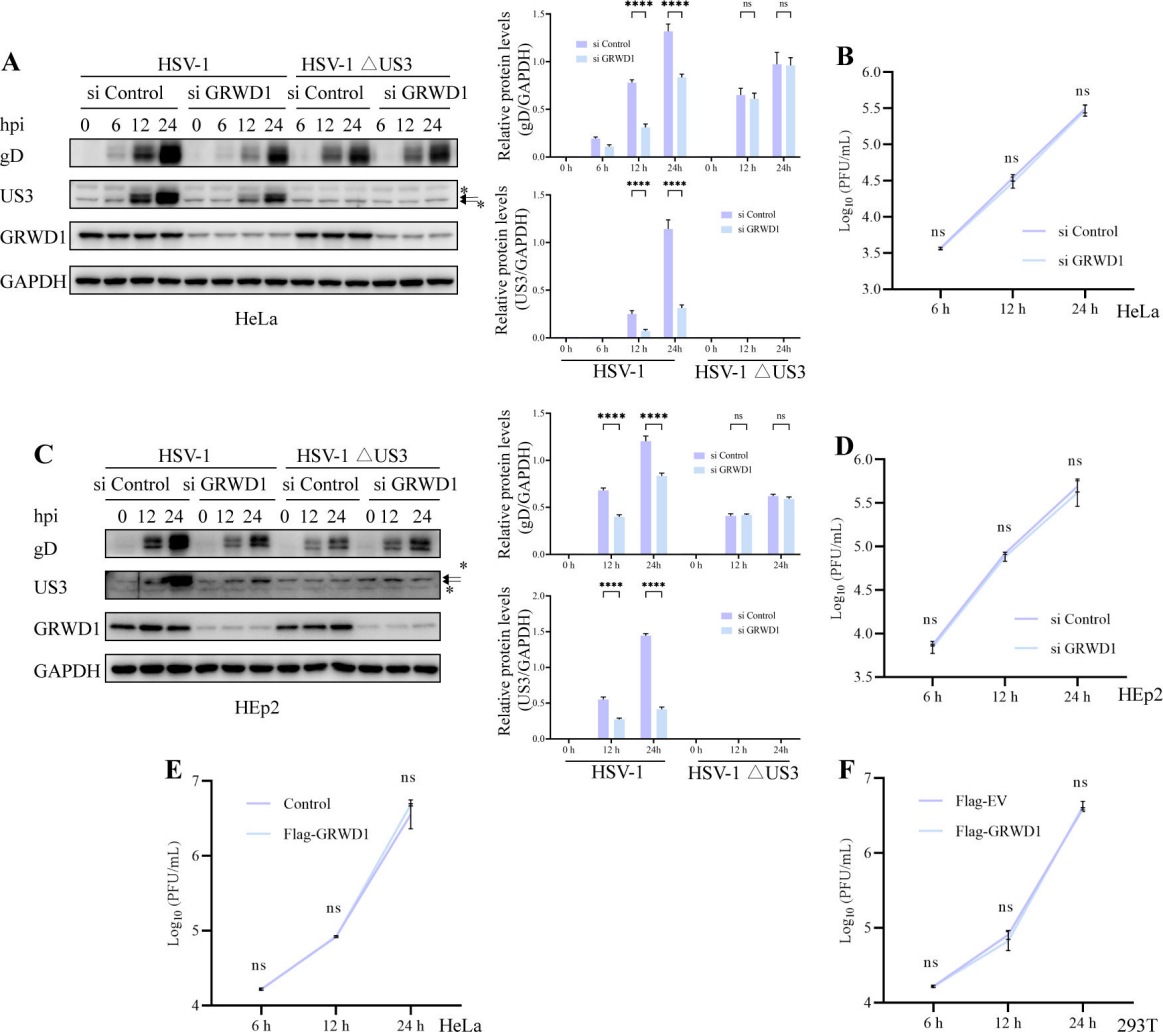
GRWD1’s role in HSV-1 replication is dependent on US3. (**A**) WB analysis of viral proteins and GRWD1 in HeLa cells transfected with si Control or si GRWD1 for 48 h, followed by infection with HSV-1 (MOI = 1) or HSV-1ΔUS3 (MOI = 1) for the indicated times. GAPDH served as a loading control. Quantification of gD and US3 band intensities (densitometric analysis by ImageJ) normalized to GAPDH is shown to the right of the WB. For US3, the major bands correspond to the two arrow-marked species; the lower band overlaps with a non-specific signal (marked by “*”). (**B**) Total infectious viral titers of HSV-1ΔUS3 in the culture of HeLa treated as described in panel **A**. (**C**) WB analysis of viral proteins in HEp2 cells treated like panel **A**. (**D**) Total infectious viral titers of HSV-1ΔUS3 in the culture of HEp2 treated as described in panel **C**. (**E**) Total infectious viral titers of HSV-1ΔUS3 in the culture of Control or Flag-GRWD1 HeLa cells at different time points. (**F**) Total infectious viral titers of HSV-1ΔUS3 in 293T cells transfected with Flag-EV or Flag-GRWD1 for 24 h and infected at the indicated times. Statistical analysis: data in panels **A–F** were analyzed by two-way ANOVA followed by Sidak’s multiple comparisons test to evaluate differences between groups at each time point. Asterisks indicate statistical significance between groups at the same time point (****, *P* < 0.0001; ns, not significant). Data are presented as mean ± SD from three independent experiments.

Since US3 partially functions in the nuclear egress of HSV-1, we next investigated whether GRWD1’s role in this process is dependent on the presence of US3. To address this, we examined the impact of GRWD1 knockdown on HSV-1ΔUS3 (US3 deletion mutant) infection. WB analysis in HeLa and HEp2 cells revealed that GRWD1 depletion selectively inhibited wild-type (WT) HSV-1 replication, as evidenced by reduced levels of viral proteins (gD and US3) but had no effect on HSV-1ΔUS3 protein expression (Fig. 3A and C). Similarly, viral titer assays demonstrated that GRWD1 knockdown impaired wild-type HSV-1 replication but did not affect HSV-1ΔUS3 replication (Fig. 3B and D). Furthermore, overexpression of GRWD1 in HeLa cells (Flag-GRWD1) and 293T cells failed to alter HSV-1ΔUS3 titers (Fig. 3E and F), reinforcing the idea that GRWD1’s function is linked to US3.

**Fig 4:**
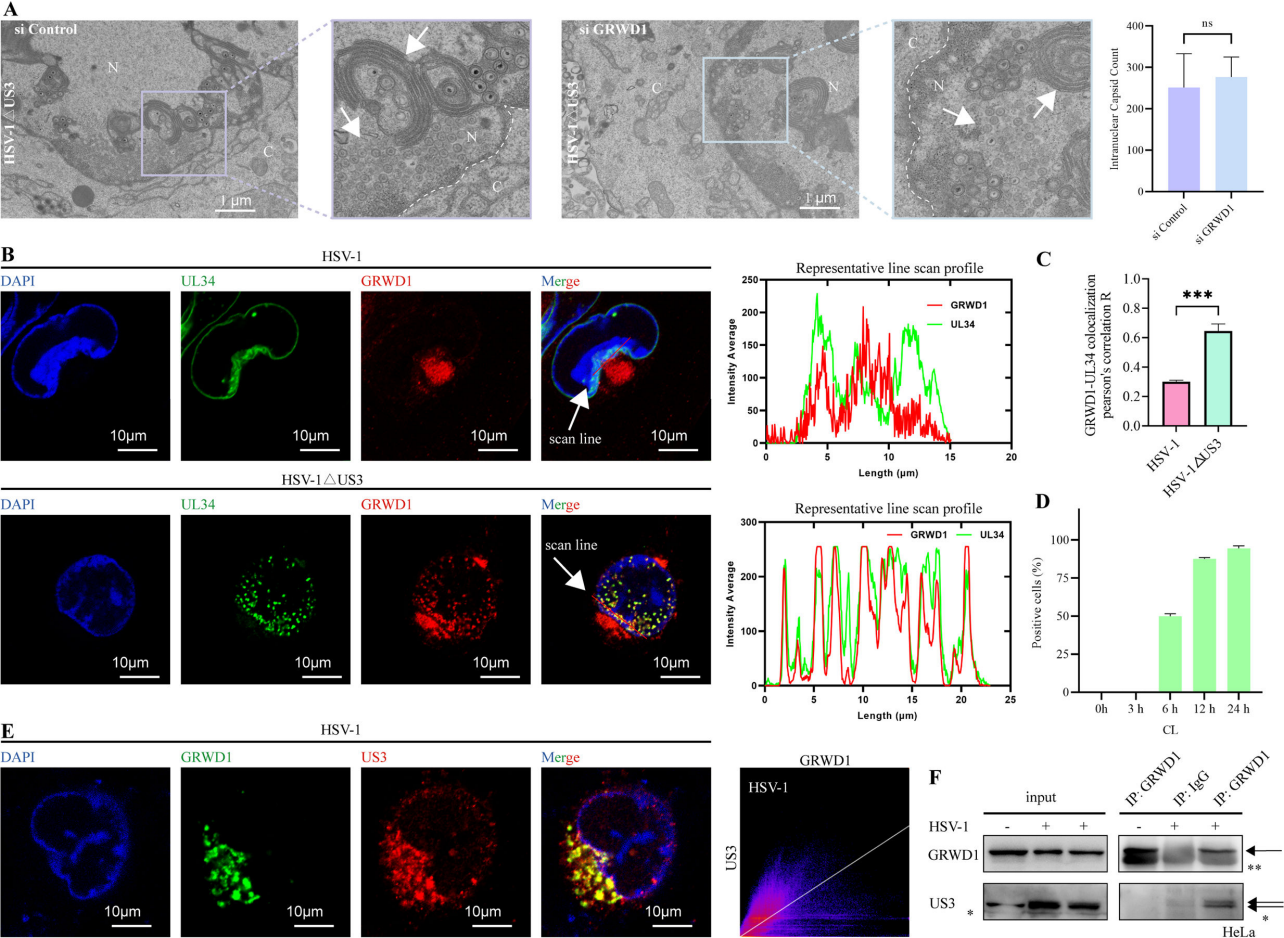
GRWD1’s role in HSV-1 nuclear egress is dependent on US3. (**A**) Representative TEM images of si Control and si GRWD1-treated HeLa cells infected with HSV-1ΔUS3 (MOI = 1, 24 hpi). N, nucleus; C, cytoplasm. Scale bars: 1 μm. Right panel: quantitative counts of nuclear capsids per nucleus from nine randomly selected fields. (**B**) Representative confocal images of HeLa cells infected with HSV-1 or HSV-1ΔUS3 (MOI = 5, 12 hpi), showing UL34 (green), GRWD1 (red), and their colocalization (merge). Scale bars: 10 μm. White arrows indicate the lines used for fluorescence intensity profiles (right panels). Line-scan intensity profiles (along the white arrows) show coincident peaks of GRWD1 and UL34 at nuclear envelope sites. (**C**) Colocalization was quantified by Pearson’s correlation coefficient (PCC) using the ImageJ Coloc2 plugin with Costes’ automatic threshold; ≥20 cells per group from three independent experiments were analyzed. The bar graph summarizes the PCC calculated from three representative groups. (**D**) Percentage of cells with GRWD1 localized in the cytoplasm (cytoplasmic localization, CL); ≥40 cells per field were analyzed across three randomly selected fields. *Y*-axis: percentage of cells (%). *X*-axis: hpi. (**E**) Representative GRWD1 (green) and US3 (red) colocalization in infected cells. PCC shown. Scale bars: 10 μm. (**F**) Co-IP of endogenous GRWD1 from HeLa cells infected with HSV-1 (MOI = 1, 24 hpi). Cells were lysed in mild lysis buffer, sonicated briefly, and incubated on ice for 30 min. IPs were performed using control IgG or anti-GRWD1-coupled Protein A/G magnetic beads; uninfected cells incubated with GRWD1-coupled beads served as an additional control. In the IP results, the primary GRWD1 band is indicated by the upper arrow, while the lower “**” denotes the heavy chain. Statistical analysis: all quantifications were tested with an unpaired two-tailed Student’s *t*-test. Data are shown as mean ± SD from three independent experiments. Significance levels: ***, *P* < 0.001; ns, not significant.

TEM analysis further supported these findings, showing that nucleocapsids accumulated within the nucleus in HSV-1ΔUS3-infected cells regardless of GRWD1-knockdown status, with the presence of nuclear membrane herniation typically observed in HSV-1ΔUS3-infected cells (Fig. 4A). This indicates that GRWD1 and US3 may act within the same pathway, with GRWD1 positioned downstream.

We also examined the distribution of GRWD1 in HSV-1-infected cells compared to HSV-1ΔUS3-infected cells by performing immunostaining. In uninfected cells, GRWD1 localized primarily to nucleoli and colocalized with fibrillarin (Fig. S1A). In HSV-1-infected cells, GRWD1 was predominantly localized in perinuclear cytoplasmic clusters, with partial colocalization with UL34 near the nuclear membrane (Fig. 4B). Upon infection, GRWD1 dispersed from the nucleolus into the nucleoplasm and, by 6 hpi, began partial nuclear-to-cytoplasmic translocation with accumulation in perinuclear regions, a timing that corresponds to established nuclear egress events (29). As infection progressed, more cells showed this redistribution, and by 12 hpi, the majority exhibited cytoplasmic perinuclear invaginations; a small fraction retained a diffuse nuclear distribution (Fig. 4D; Fig. S1B and D).

In contrast, in HSV-1ΔUS3-infected cells, GRWD1 largely colocalized with UL34 in nuclear punctate structures, while only a minor fraction redistributed to perinuclear cytoplasmic aggregates (Fig. 4B). Line-scan intensity profiles (along red paths indicated by white arrows) demonstrated that in HSV-1-infected cells, GRWD1 (red) and UL34 (green) fluorescence exhibited only sporadic coincident peaks near the nuclear envelope (typical line length 5–10 μm; representative traces shown). By contrast, HSV-1ΔUS3-infected cells showed multiple coincident peaks of GRWD1 and UL34 intensity in the same region, consistent with enhanced punctate colocalization foci within the nucleus (Fig. 4B). This further demonstrates the interconnection between nuclear egress and GRWD1, as their colocalization suggests a potential association of GRWD1 with nuclear egress complexes, although its exact functional role requires clarification. Additionally, impaired nuclear egress concurrently restricted partial cytoplasmic translocation of GRWD1. The nuclear puncta may represent impaired nuclear egress complexes. Co-staining with UL34 confirmed that UL34 localizes smoothly along the nuclear membrane in HSV-1-infected cells. The observed UL34 localization pattern represents a typical phenotype during wild-type HSV-1 infection, potentially linked to efficient nuclear egress (30). Quantitative analysis using PCC confirmed significantly stronger GRWD1-UL34 colocalization in HSV-1ΔUS3-infected cells (mean PCC ≈ 0.63) versus HSV-1 controls (mean PCC ≈ 0.3) (Fig. 4C; Fig. S1C). Intriguingly, this inverse correlation between US3 presence and colocalization efficiency suggests that US3 may function as a negative regulator of GRWD1-UL34 spatial interactions.

Given the strong functional association between GRWD1 and US3 in positively regulating HSV-1 replication and nuclear egress processes, we further examined their colocalization during infection. Fluorescence imaging revealed robust codistribution of US3 and GRWD1 perinuclear cytoplasmic clusters (Fig. 4E). Quantitative PCC analysis confirmed high-level colocalization between GRWD1 and US3 (mean PCC ≈ 0.8). To validate physical interaction, Co-IP assays were performed. In HSV-1-infected HeLa cells, endogenous GRWD1 was found to interact with US3 (Fig. 4F). In Flag-GRWD1 stably overexpressing HeLa cells (Flag-GRWD1), Flag-GRWD1 also interacted with US3 (Fig. S1E). These results collectively demonstrate that GRWD1 specifically interacts with US3.

Collectively, these data demonstrate that GRWD1-dependent regulation of HSV-1 replication is contingent on the presence of US3, indicating a cooperative interaction between this host factor and the viral kinase in facilitating nuclear egress.

### GRWD1 interacts with Lamin A/C and regulates its expression during HSV-1 infection

**Fig 5:**
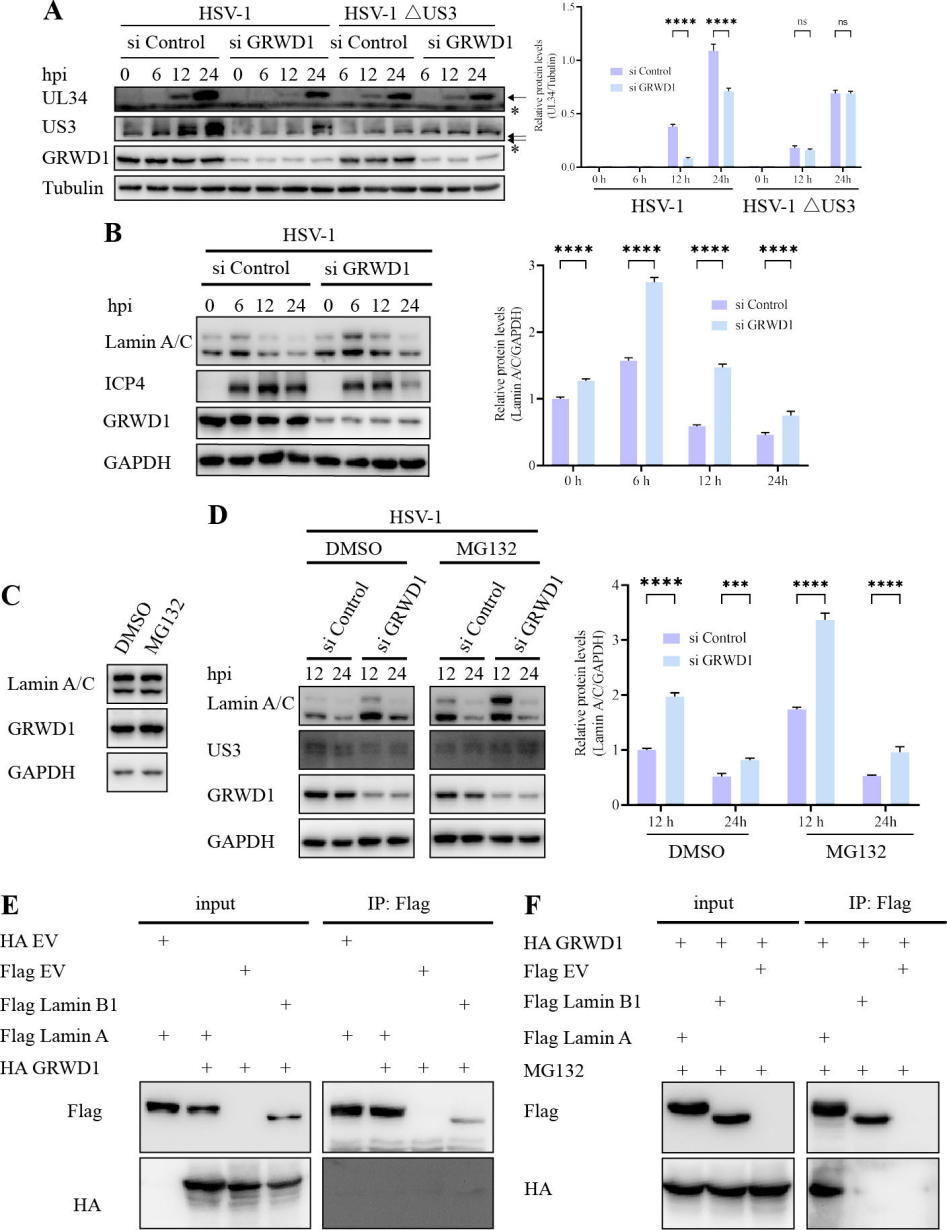
GRWD1 interacts with Lamin A/C and regulates its expression during HSV-1 infection. (**A**) WB analysis of viral proteins and GRWD1 in HeLa cells transfected with si Control or si GRWD1 for 48 h, followed by infection with HSV-1 or HSV-1ΔUS3 (MOI = 1). Tubulin serves as a loading control. The UL34 band was normalized to tubulin, with quantitative data shown graphically to the right of the WB. Arrow: UL34-specific band; *: non-specific band. (**B**) WB analysis of Lamin A/C, ICP4, and GRWD1 in HeLa cells treated as in panel **A** and infected with HSV-1 (MOI = 1) at the indicated times. GAPDH served as the loading control. Right: quantification of Lamin A/C normalized to GAPDH. (**C**) Lamin A/C analysis in uninfected cells with proteasome inhibition. HeLa cells were left uninfected and treated with or without MG132 (10 μM, added 4 h prior to harvest). Lamin A/C and GRWD1 were detected by WB, with GAPDH serving as the loading control. (**D**) Lamin A/C analysis with proteasome inhibition: Cells were treated as in panel **B** with or without MG132 (10 μM, added 4 h prior to harvest). (**E and F**) Co-IP analysis. Flag-tagged Lamin A, Lamin B1, or EV was immunoprecipitated using anti-Flag magnetic beads, and co-precipitated GRWD1 was detected by anti-HA immunoblotting. Statistical analysis: data in panels **A–D** were analyzed by two-way ANOVA followed by Sidak’s multiple comparisons test to evaluate differences between groups at each time point. Asterisks denote significance between groups at the same time point (***, *P* < 0.001; ****, *P* < 0.0001; ns, not significant). Data are presented as mean ± SD from three independent experiments.

To investigate the mechanism by which GRWD1 regulates HSV-1 nuclear egress, we first examined whether GRWD1 knockdown directly affects the NEC. UL34, a core component of the NEC, is essential for nuclear egress, as its deficiency blocks this process (31). Results showed that GRWD1 knockdown did not alter UL34 protein levels in HSV-1ΔUS3-infected cells (Fig. 5A), suggesting that GRWD1 depletion may not directly impair NEC function. Although reduced UL34 levels were observed upon GRWD1 knockdown during WT HSV-1 infection, this effect likely arose from secondary consequences of multi-round viral replication. Given that the nuclear lamina serves as a structural barrier to nucleocapsid-nuclear envelope contact prior to nuclear egress, we explored the potential role of GRWD1 in modulating Lamin proteins. Notably, US3 has been shown to phosphorylate Lamins and alter their subcellular localization (19), though its direct involvement in Lamin destabilization or degradation remains to be fully elucidated. Given GRWD1’s potential role as a substrate receptor for E3 ubiquitin ligases, we hypothesized that GRWD1 may facilitate the proteasomal degradation of Lamins. To test this hypothesis, we first examined whether the protein levels of Lamin A/C changed during HSV-1 infection in HeLa cells with or without GRWD1 knockdown. WB analysis revealed that Lamin A/C increased at 6 hpi but declined at 12 and 24 hpi in both control and GRWD1-knockdown cells. However, GRWD1-knockdown cells consistently exhibited higher Lamin A/C levels compared to control cells across all time points examined (Fig. 5B). To determine if this reduction involves the proteasome, we treated cells with the proteasome inhibitor MG132. Notably, MG132 treatment did not alter Lamin A/C levels in uninfected cells (Fig. 5C), suggesting that basal turnover of Lamin A/C is minimal in this context. In contrast, MG132 treatment of infected cells at 12 hpi markedly increased Lamin A/C protein levels, particularly for Lamin A (Fig. 5D), indicating that Lamin degradation is specifically induced during HSV-1 infection. Co-immunoprecipitation assays revealed that exogenous HA-GRWD1 interacted with Flag-tagged Lamin A but not Lamin B1, and this interaction was detectable only in the presence of MG132 (Fig. 5E and F). This indicates that the Lamin A species capable of binding GRWD1 are normally targeted for proteasomal degradation.

Taken together, these results suggest that Lamin A/C is transiently upregulated early during infection, potentially as part of a host defense mechanism, but is subsequently targeted for proteasomal degradation at later stages of infection. GRWD1 appears to play a role in facilitating this virus-induced degradation of Lamin A/C, thereby contributing to the modulation of nuclear lamina dynamics and promoting efficient nuclear egress during HSV-1 infection.

## DISCUSSION

Our study identifies GRWD1 as a novel host factor that facilitates HSV-1 nuclear egress, a pivotal step in capsid transit from the nucleus to the cytoplasm. Mechanistically, GRWD1 promotes the proteasomal degradation of Lamin A/C. While US3 interacts with GRWD1 and GRWD1-mediated HSV-1 replication depends on US3, whether US3 directly contributes to GRWD1’s role in Lamin A/C degradation requires further investigation. During HSV-1 infection, GRWD1 undergoes dynamic relocalization, shifting from nucleoli to perinuclear cytoplasmic clusters, a spatial redistribution that appears functionally linked to nuclear egress progression. These coordinated alterations reveal intricate host-virus interactions.

The observation that nucleocapsids were trapped in the nucleus in GRWD1-knockdown cells is striking, indicating that GRWD1 plays an active role in facilitating nuclear egress. This process is initiated by NEC proteins UL31 and UL34, requiring nucleocapsids to reach the nuclear envelope’s vicinity and interact with the NEC. As a histone-binding protein regulating chromatin dynamics, GRWD1 may enable nucleocapsids to access the nuclear rim by modulating chromatin status (21), potentially creating a reported chromatin-thin tunnel. Alternatively, interaction with the DDB1-CUL4 ligase could ubiquitinate host proteins (27), such as lamina components, thereby aiding egress. Studies have demonstrated that the expansion of viral replication compartments following HSV-1 infection is accompanied by nuclear enlargement (32). This phenomenon may be attributed to the upregulation of Lamin A/C protein levels during the early stages of infection, which could exert inhibitory effects on the nuclear egress of viral nucleocapsids. Notably, the nuclear egress process typically initiates after 6 hpi (29). Our results suggest that HSV-1 hijacks GRWD1 to promote Lamin A/C degradation at this stage, facilitating efficient nuclear egress of progeny virions. Importantly, this GRWD1-dependent egress is tightly linked to US3: HSV-1ΔUS3 shows abnormal nuclear envelope morphology and perinuclear capsid accumulation (33, 34), and in our study, GRWD1 effects on egress were abolished in the absence of US3. This suggests that GRWD1 may act downstream of US3, potentially as a phosphorylation target or co-factor enhancing NEC function. Moreover, GRWD1’s relocalization from the nucleolus to perinuclear clusters during infection further supports its involvement in coordinating nuclear and cytoplasmic phases of egress, though whether this occurs via nuclear pores or through poorly understood outer nuclear membrane remodeling remains to be clarified.

GRWD1 could be involved in HSV-1 replication primarily through nuclear egress-associated processes. During late-phase infection, knockdown of GRWD1 was associated with reduced viral propagation, particularly under conditions that favor sustained multi-round replication. The time-dependent and MOI-dependent kinetics observed in viral genome quantification suggest that the impact of GRWD1 becomes evident during ongoing cycles of infection rather than during the very early events of viral entry or genome replication. Moreover, the discrepancy between intracellular viral DNA levels and extracellular levels in the culture supernatant indicates that GRWD1 may influence steps related to nuclear egress, capsid transit, and subsequent viral spread, rather than directly modulating viral DNA synthesis. These observations collectively support a role for GRWD1 in processes functionally linked to nuclear egress, while additional contributions to other stages of the viral life cycle remain possible. In addition to nuclear egress-related mechanisms, GRWD1 may also exert indirect effects through host regulatory pathways. The tumor suppressor protein p53 has been reported to enhance HSV-1 and PRV replication (35, 36). During early HSV-1 infection, p53 upregulates ICP27 expression (36). ICP27 is a multifunctional protein and has been reported to regulate multiple steps of mRNA synthesis and processing, including transcription, splicing, and nuclear export (37). Intriguingly, although GRWD1 has been shown to negatively regulate p53 (25, 26), we observed that GRWD1 knockdown in HeLa cells suppressed p53 levels (data not shown), which might reduce ICP27 expression and thereby inhibit HSV-1 transcriptional activity. However, HeLa cells are not an ideal system for studying p53 due to their instability (38). Thus, whether GRWD1 affects HSV-1 transcription via p53 requires further investigation in more suitable models. Additionally, ADAM metallopeptidase domain 17 (ADAM17), which is regulated by GRWD1 (39), has also been implicated in herpesvirus replication (40), raising the possibility that GRWD1 might modulate HSV-1 through ADAM17—a hypothesis that requires systematic validation.

We acknowledge the limitations of the current study. Although our data consistently support a role for GRWD1 in nuclear egress, they do not yet fully resolve whether GRWD1’s principal function in this context is mechanistic (direct remodeling of nuclear lamina architecture) or regulatory (modulation of factors that in turn alter lamina stability or transcription). Additionally, while our imaging and biochemical data are reproducible and internally consistent, more extensive quantitative population-level assays (for example, larger-scale cell sampling for colocalization metrics, targeted ubiquitin profiling, or time-resolved RNA analyses) would strengthen causal inference and are appropriate next steps.

## MATERIALS AND METHODS

### Plasmids and primers

The PRK5-Flag/HA empty vector, pSIN-Flag-empty vector, psPAX2, and pMD2.G plasmids are stored in our laboratory. In this study, plasmid construction was performed as follows: first, target DNA fragments were amplified by PCR using specific primers: Flag-GRWD1 (forward primer: 5′-AAGGACGACGATGACAAGGGATCCATGGCGGCGCGCAAGGGT-3′, reverse primer: 5′-AGTTGGGCCATGGCGGCCAAGCTTTCAGACGCTGATGGTGCGGAA-3′), HA-GRWD1 (forward primer: 5′-GACTATGCGGGCGGATCCATGGCGGCGCGCAAGGGT-3′, reverse primer: same as Flag-GRWD1 reverse primer), Flag-Lamin A (forward primer: 5′-AAGGACGACGATGACAAGGGATCCATGGAGACCCCGTCCCAG-3′, reverse primer: 5′-AGTTGGGCCATGGCGGCCAAGCTTTTACATGATGCTGCAGTT-3′), Flag-Lamin B1 (forward primer: 5′-AAGGACGACGATGACAAGGGATCCATGGCGACTGCGACCCCC-3′, reverse primer: 5′-AGTTGGGCCATGGCGGCCAAGCTTTTACATAATTGCACAGCT-3′), pSIN-Flag-GRWD1 (forward primer: 5′-GATGACGATGACGACAAGGAATTCATGGATTACAAGGATGACGAC-3′, reverse primer: 5′-TGCATGCGGATCCTTCGAACTAGTTCAGACGCTGATGGTGCGGAA-3′). Subsequently, new plasmids were rapidly assembled using homologous recombination.

### Cell culture and transfection

HeLa, HEp2, and Vero E6 cells were cultured in Dulbecco’s modified Eagle medium supplemented with 10% fetal bovine serum and 1% penicillin/streptomycin at 37°C in a 5% CO_2_ atmosphere. These cell lines are maintained in our laboratory. Stably overexpressing Flag-GRWD1 (Flag-GRWD1) or Flag-empty vector (Control) HeLa cell lines were established by the author. Briefly, 293T cells were co-transfected with the pSIN-Flag GRWD1 plasmid (or pSIN-Flag-empty vector as control), along with psPAX2 and pMD2.G plasmids, to produce lentiviral particles. The harvested lentivirus was then used to infect HeLa cells, followed by monoclonal screening to isolate stable transductants. All cell cultures were maintained under standard conditions as previously described. Cells were transfected with specific siRNAs targeting GRWD1 (si GRWD1, 5′-GGCUCCUGAGCAGCAAUAAAGGATT-3′) or control siRNA (si Control, 5′-UUCUCCGAACGUGUCACGUTT-3′) using Lipofectamine RNAiMAX transfection reagent (Invitrogen) according to the manufacturer’s protocol. For knockdown experiments, cells were initially seeded in 6-well plates and allowed to grow to approximately 40% confluence before transfection. Following overnight incubation for knockdown efficiency, cells were trypsinized and re-seeded into 12-well plates. After an additional 24 h incubation period, when cell density reached 3 × 10^5^ cells/mL, viral infection or other subsequent experiments were performed. This standardized protocol ensured consistent cell numbers across all experimental replicates while maintaining optimal growth conditions throughout the procedure. In contrast, knockdown of UL34 (si UL34, #1: 5′-GGUGCGCCUUUCAGUUUCATT-3′; #2: 5′-CUGCGGCUUAUGAACGACUTT-3′) was performed concurrently with viral infection. For overexpression experiments, plasmids were transfected into different cells using Lipofectamine LTX reagent and PLUS reagent (Invitrogen).

### Antibodies and reagents

Anti-US3 antibody was described previously (28). Antibodies against ICP0 (ab6513), ICP4 (ab6514), and gD (ab6507) were purchased from Abcam; GRWD1 (10354-1-AP) and GAPDH (60004-1-Ig) from Proteintech Group; α-tubulin (PM054) from MBL Biotechnology; Lamin A/C (AF7350) from Beyotime Biotechnology; the anti-UL34 were generated by the authors through immunizing mice with a truncated UL34 protein lacking the transmembrane domain, followed by serum collection. Fluorescent secondary antibodies for immunofluorescence assay (IFA) were purchased from Dingguo Biotechnology. Antibody dilutions: 1:1,000 for western blot and 1:200 for immunofluorescence. MG132 (10 μM), PAA (200 ng/mL), Protein A/G magnetic beads, and anti-Flag magnetic beads were purchased from MedChemExpress (MCE). One-step Cloning Kit (based on homologous recombination) was obtained from Genesand Biotechnology.

### Viruses and viral titer measurement

The KOS stain of HSV-1 and HSV-1 US3-knockout virus (HSV-1ΔUS3) were stored in our lab (28, 41). Viral titer was measured by plaque assay via Vero E6 cells. Briefly, Vero E6 cells were infected with serial virus dilutions and overlaid with low-melting-point agarose. After 3 days of incubation at 37°C, cells were fixed with paraformaldehyde for 6 h, stained with 0.01% crystal violet for 15 min, followed by washing and plaque counting. Plaque-forming units were determined.

### Co-immunoprecipitation

Whole-cell lysates were collected and lysed in either mild or strong lysis buffer. The lysis buffer formulations used in this study were as follows: mild lysis buffer: Tris-Cl (1 M, pH 7.4), NaCl (1 M), NP-40, EDTA (0.5 M, pH 8.0), and glycerol. Strong lysis buffer (MCE): 50 mM Tris (pH 7.4), 150 mM NaCl, 1% Triton X-100, 1% sodium deoxycholate, 0.1% SDS, sodium orthovanadate, and EDTA. Prior to use, all lysis buffers were supplemented with 1% protease inhibitor cocktail (MCE). For mild lysis buffer-treated samples, brief sonication was performed during cell harvesting. After incubating the lysates at 4°C for 30 min, the samples were centrifuged at 13,000 rpm for 10 min. A portion of the supernatant was collected as Input. The remaining lysate was incubated with Flag-tagged magnetic beads at room temperature for 30 min, followed by 4°C for 2 h. Finally, the beads were washed three times with the corresponding lysis buffer (mild or strong), and the resulting sample was designated as IP.

### WB

Cells were lysed in RIPA buffer with protease inhibitors, and proteins were separated by SDS-PAGE and transferred to a PVDF membrane. Membranes are blocked and incubated with primary antibodies, followed by HRP-conjugated secondary antibodies. Bands were visualized via chemiluminescence (ECL) luminometer using the Immobilon Classico Western HRP substrate (Millipore).

### Quantitative RT-PCR

Total RNA was extracted with HiPure Total RNA Mini Kit (Magen Biotechnology). A reverse transcription (RT) system (Promega) was used to synthesize cDNA. Total DNA was extracted with Universal Genomic DNA Purification Mini Spin Kit (Beyotime). SYBR green PCR mix (CWBiotech) and a real-time PCR system (Roche 480) were used for quantitative RT-PCR.

### IFA

Cells on coverslips were fixed with 4% paraformaldehyde, permeabilized with 0.2% Triton X-100 (Sigma-Aldrich), and blocked with 2% BSA. After incubation with primary antibodies, fluorescent secondary antibodies were applied. Nuclei were stained with DAPI, and images were captured via fluorescence or confocal microscopy (42).

### TEM

HeLa cells were infected with HSV-1 or HSV-1ΔUS3 (MOI = 1, 24 h), fixed with 1% paraformaldehyde and 2.5% glutaraldehyde, post-fixed in 1% osmium tetroxide, and dehydrated through an acetone gradient. After embedding in Spurr’s resin, ultrathin sections (60–80 nm) were cut, stained with uranyl acetate/lead citrate, and imaged by TEM (Hitachi HT7800).

## SUPPLEMENTAL MATERIAL

Fig. S1 spectrum.01608-25-s0001.tif HSV-1 genome copy numbers in cells and culture supernatants after GRWD1 knockdown.

Fig. S2 spectrum.01608-25-s0002.tif GRWD1's role in HSV-1 nuclear egress is dependent on US3.

Supplemental material spectrum.01608-25-s0003.docx Supplemental figure legends.

ASM does not own the copyrights to Supplemental Material that may be linked to, or accessed through, an article. The authors have granted ASM a non-exclusive, world-wide license to publish the Supplemental Material files. Please contact the corresponding author directly for reuse.
