# Supplementary figures and images for: GRWD1 enhances HSV-1 replication by facilitating nuclear egress
Source: Microbiol Spectr. 2026 Apr 17;14(6):e01608-25. doi: 10.1128/spectrum.01608-25 (PMC13228041; doi:10.1128/spectrum.01608-25)

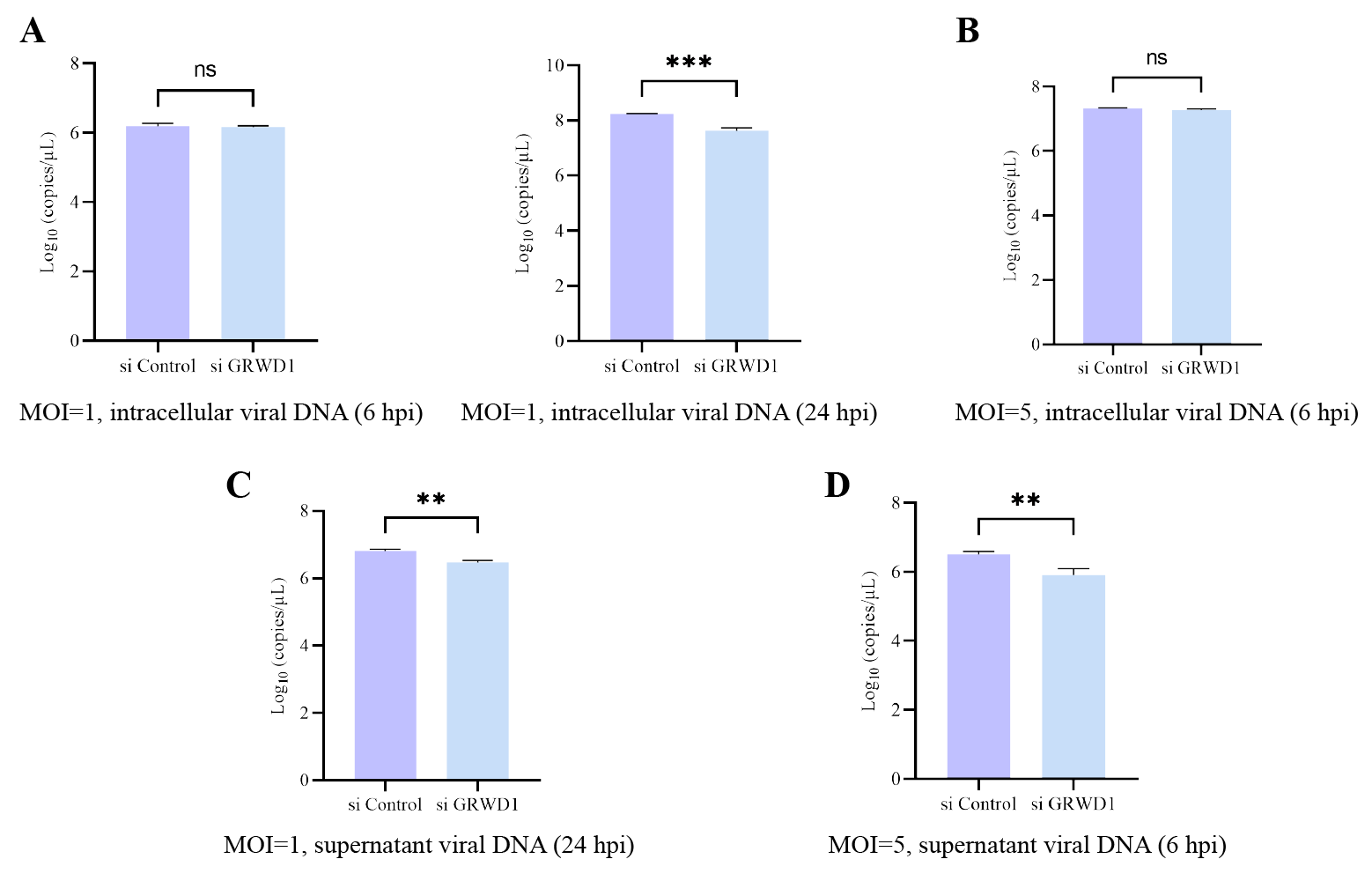

Supplement: Fig. S1 — HSV-1 genome copy numbers in cells and culture supernatants after GRWD1 knockdown. [file spectrum.01608-25-s0001.tif]

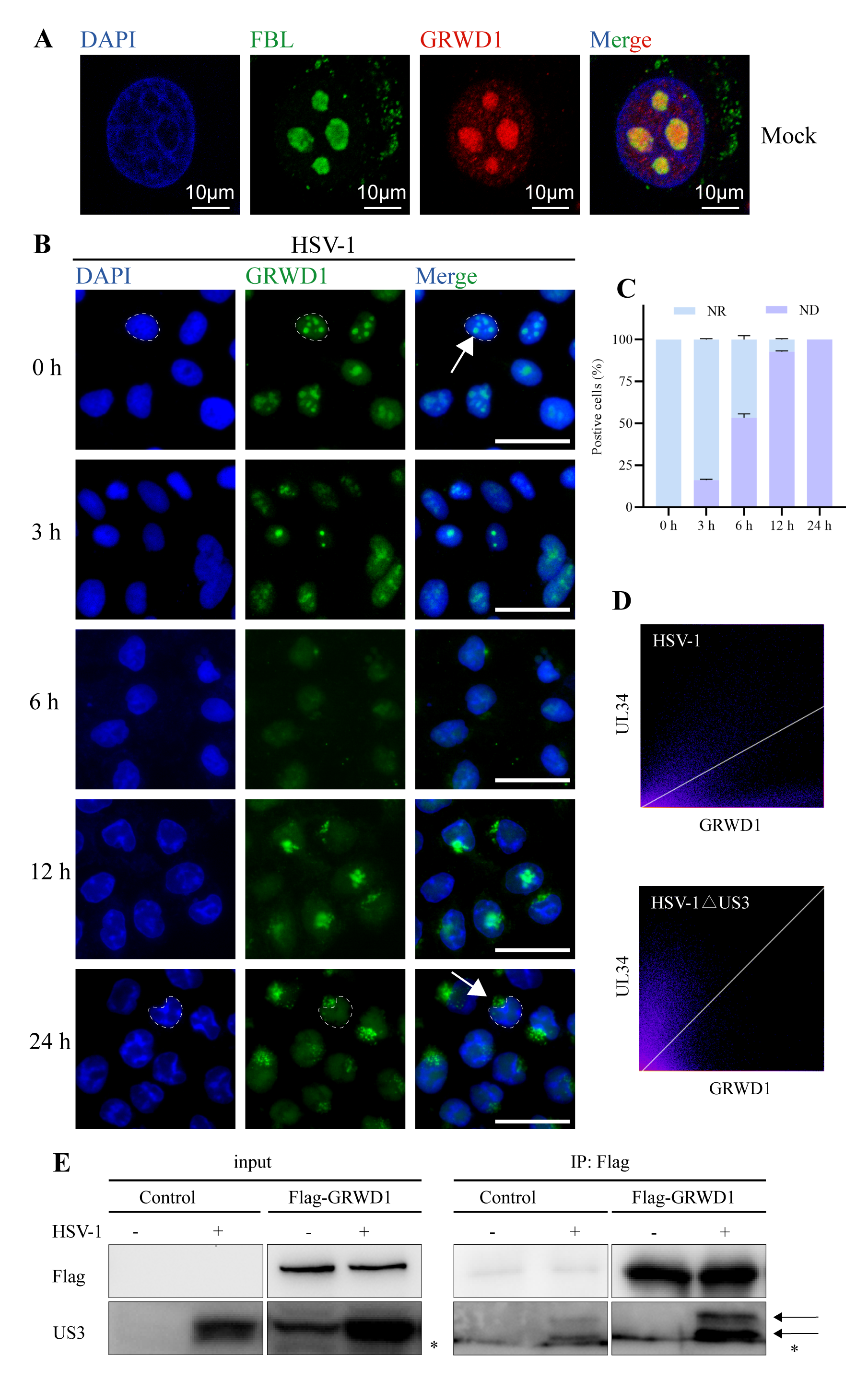

Supplement: Fig. S2 — GRWD1's role in HSV-1 nuclear egress is dependent on US3. [file spectrum.01608-25-s0002.tif]
