# Supplementary material for: GRWD1 enhances HSV-1 replication by facilitating nuclear egress
Source: Microbiol Spectr. 2026 Apr 17;14(6):e01608-25. doi: 10.1128/spectrum.01608-25 (PMC13228041; doi:10.1128/spectrum.01608-25)
Supplement: Supplemental material — Supplemental figure legends. [file spectrum.01608-25-s0003.docx]

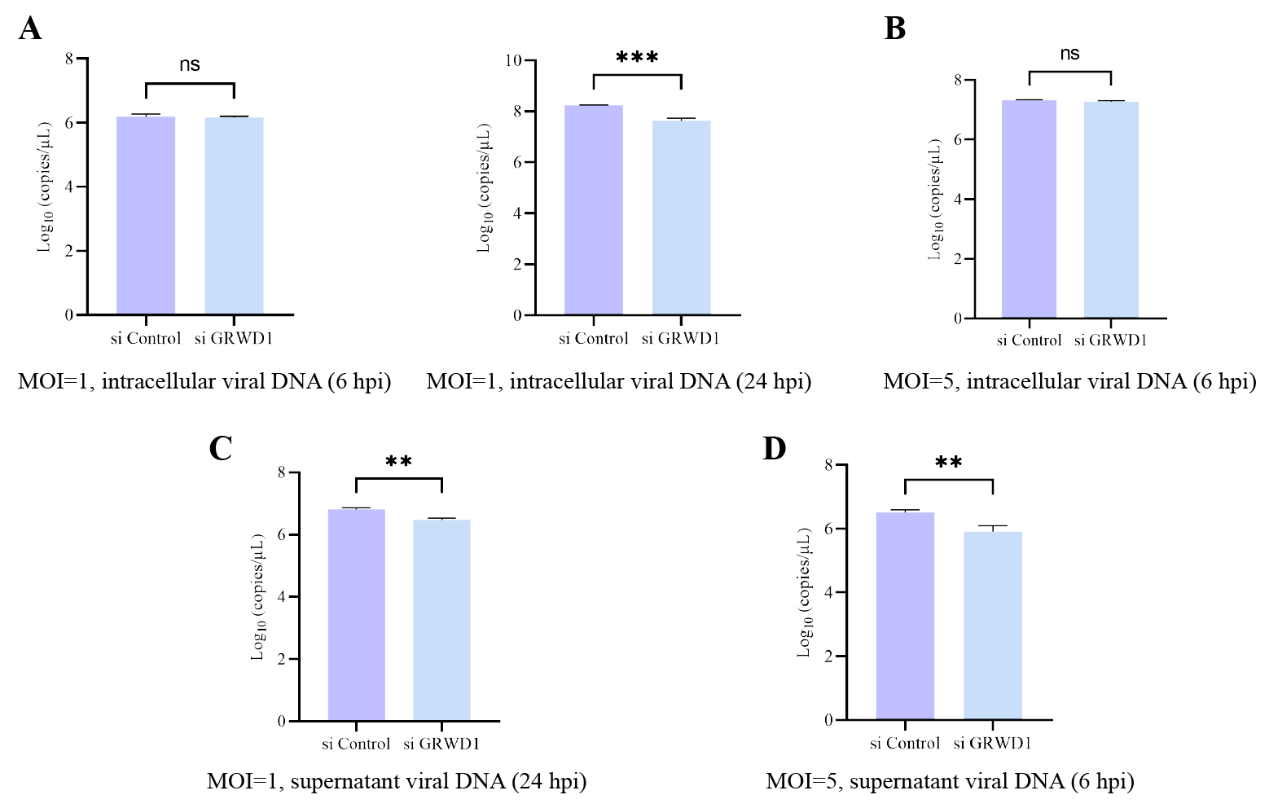


FIG S1 HSV-1 genome copy numbers in cells and culture supernatants after GRWD1 knockdown. (A) Viral genome copy numbers in cell lysates from HeLa cells infected with HSV-1 at an MOI=1 and harvested at 6 hpi or 24 hpi after si Control or si GRWD1 treatment. (B) Viral genome copy numbers in cell lysates from HeLa cells infected with HSV-1 at MOI=5 and harvested at 6 hpi after si Control or si GRWD1 treatment. (C) Viral genome copy numbers in culture supernatants from HeLa cells infected with HSV-1 at an MOI=1 and harvested at 24 hpi after si Control or si GRWD1 treatment. (D) Viral genome copy numbers in culture supernatants from HeLa cells infected with HSV-1 at MOI=5 and harvested at 6 hpi after si Control or si GRWD1 treatment. Viral DNA was quantified by qPCR. Data are presented as the mean ± SD from three independent experiments. Statistical analysis: unpaired two-tailed Student’s t test. Significance levels: *, P < 0.05; **, P < 0.01; ***, P < 0.001; ns, not significant.


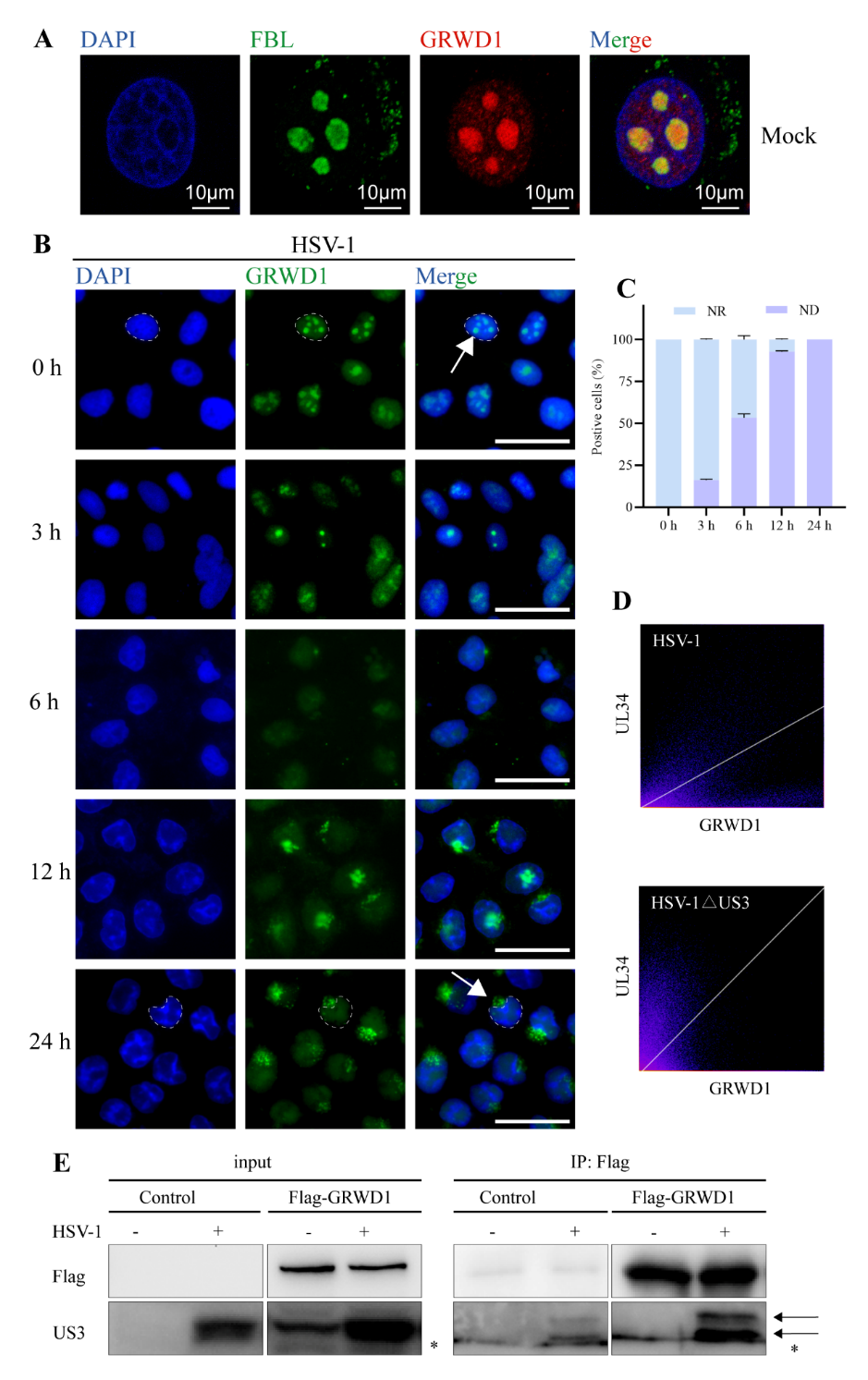


FIG S2 GRWD1's role in HSV-1 nuclear egress is dependent on US3. (A) Confocal images of HeLa cells, showing FBL (green), GRWD1 (red), and their colocalization (merge). Scale bars: 10 μm. (B) IFA analysis of GRWD1 (green) in HeLa cells infected with HSV-1 (MOI=5) at 0, 3, 6, 12 and 24 hpi, with DAPI-stained nuclei (blue). Scale bars: 50 μm. White arrows: GRWD1 localization, white dashed outlines: Nuclei. (C) Percentage of cells with GRWD1 localized in intact nucleoli (nucleolar retention, NR) versus dispersed from disintegrating nucleoli (nucleolar dispersion, ND), quantified from IFA images, ≥40 cells per field were analyzed across three randomly selected fields. Y-axis: percentage of cells (%). X-axis: hpi. (D) Colocalization was quantified by Pearson’s correlation coefficient (PCC) using the ImageJ Coloc2 plugin with Costes’ automatic threshold. (E) Control or Flag-GRWD1 HeLa cells infected with HSV-1 (MOI=1, 24 hpi) were lysed with stringent lysis buffer, subjected to 30-min ice-cold lysis, and incubated with Flag magnetic beads.
